# Efficacy of Glycyrrhetinic Acid in the Treatment of Acne Vulgaris Based on Network Pharmacology and Experimental Validation

**DOI:** 10.3390/molecules29102345

**Published:** 2024-05-16

**Authors:** Lingna Xie, Congwei Ma, Xinyu Li, Huixiong Chen, Ping Han, Li Lin, Weiqiang Huang, Menglu Xu, Hailiang Lu, Zhiyun Du

**Affiliations:** 1 School of Biomedical and Pharmaceutical Sciences, Guangdong University of Technology, Guangzhou 510006, China; 1112012007@mail2.gdut.edu.cn (L.X.); 2112112026@mail2.gdut.edu.cn (C.M.); huixiong.chen@parisdescartes.fr (H.C.); 2 Shenzhen Liran Cosmetics Co., Ltd., Shenzhen 518000, Chinahwq@makesensegroup.com (W.H.); menglu.xu@makesensegroup.com (M.X.); 3 Chemistry of RNA, Nucleosides, Peptides and Heterocycles, CNRS UMR8601, Université Paris Cité, 45 Rue des Saints-Pères, CEDEX 06, 75270 Paris, France; 4 Foshan Allan Conney Biotechnology Co., Ltd., Foshan 528231, China; ping.han@allan-conney.com (P.H.); annie.lin@allan-conney.com (L.L.)

**Keywords:** acne vulgaris, Glycyrrhetinic acid, network pharmacology, molecular docking, TMT, pro-inflammatory cytokines

## Abstract

Glycyrrhetinic acid (GA) is a saponin compound, isolated from licorice (Glycyrrhiza glabra), which has been wildly explored for its intriguing pharmacological and medicinal effects. GA is a triterpenoid glycoside displaying an array of pharmacological and biological activities, including anti-inflammatory, anti-bacterial, antiviral and antioxidative properties. In this study, we investigated the underlying mechanisms of GA on acne vulgaris through network pharmacology and proteomics. After the intersection of the 154 drug targets and 581 disease targets, 37 therapeutic targets for GA against acne were obtained. A protein–protein interaction (PPI) network analysis highlighted TNF, IL1B, IL6, ESR1, PPARG, NFKB1, STAT3 and TLR4 as key targets of GA against acne, which is further verified by molecular docking. The experimental results showed that GA inhibited lipid synthesis in vitro and in vivo, improved the histopathological damage of skin, prevented mast cell infiltration and decreased the level of pro-inflammatory cytokines, including TNF-α, IL-1β and IL-6. This study indicates that GA may regulate multiple pathways to improve acne symptoms, and the beneficial effects of GA against acne vulgaris might be through the regulation of sebogenesis and inflammatory responses.

## 1. Introduction

Acne vulgaris is a persistent, cutaneous and inflammatory disorder of pilosebaceous follicles, which is characterized by clinical manifestations of papules, pustules or nodules, seborrhea, inflammatory lesions and various degrees of scarring due to cyst formation [1]. The epidemiology of acne vulgaris has revealed the prevalence in teenagers is more than 60%, which is the highest prevalence of all ages [2]. Although it remains unknown, the main acne pathogenesis has been identified to be related to an abundance of *Propionibacterium acnes* (*P. acnes*), high androgen levels, excessive secretion of sebum and hair follicle hyperkeratosis. The interaction of these pathogenic factors results in the formation of comedones and the development of inflammation [3]. Androgen excess can cause acne by triggering sebaceous glands (SGs) in the skin to overproduce sebum, which clogs pores and hair follicles, influences inflammation of the skin and feeds *P. acnes*, thereby resulting in acne vulgaris [4]. SGs are composed of sebocytes, which produce different pro-inflammatory cytokines (TNF-α, IL-1β, IL-6, IL-8), accumulate lipids and release the lipid-containing sebum [5]. Sebaceous gland activity is a driver of inflammation in acne vulgaris. The dysfunction of SGs has been closely associated with a variety of skin diseases, such as seborrheic dermatitis, psoriasis, rosacea and acne [6]. 

The function of sebum is involved in maintaining part of the epidermal barrier and the immune system of the skin [7]. *P. acnes* is a non-spore-forming, Gram-positive bacterium, found in sebaceous follicles of human skin. *P. acnes* modulates keratinocyte differentiation by inducing integrin and filaggrin expression and aggravates local inflammation, thereby resulting in early-stage acne microcomedones and inflammatory lesions in acne vulgaris [8]. Furthermore, *P. acnes* promotes SGs, lipogenesis and sebum lipogenesis through the corticotropin-releasing hormone receptor (CRFR) signaling pathway [9].

Antibiotics targeting *P. acnes* have always been an efficient therapy for treating acne vulgaris [10]. However, antibiotic resistance raises increasing concerns in clinical practice because of long-term antibiotic use. Antibiotics are not suitable to be used for maintenance therapy in acne treatment. Therefore, searching for new bioactive compounds derived from plants and other sources with high therapeutic efficiency and low adverse effects is necessary, and this will offer great hope for finding new drugs for treating acne vulgaris.

Glycyrrhizin (Glycyrrhizic acid) is the chief sweet-tasting and water-soluble constituent of licorice root. Structurally, it is a triterpenoid saponin glycoside, which is used as a gel-forming and emulsifying agent in foodstuffs and cosmetics. Licorice root extract has important applications in traditional medicine and has been wildly explored for its intriguing pharmacological and medicinal effects in treating various diseases, such as respiratory and urinary tract infections, indigestion, gastritis, peptic ulcers, expulsion of kidney stones and acne vulgaris [11]. It is also found that licorice flavonoids play an anti-acne role by regulating the metabolic balance related to inflammation, sebum overflow and follicular keratosis as well as microbial balance [12]. In particular, Glycyrrhetinic acid (GA), the major metabolic product of glycyrrhizic acid, has attracted widespread attention due to its various pharmacological effects, including anti-inflammatory, anti-bacterial and antioxidative activities [13,14,15]. Moreover, it also has many applications in cosmetology and dermatology, including hyperpigmentation, atopic dermatitis, excessive fat, androgenetic alopecia and skin aging, with few side effects [16,17]. Recently, Fang-Ning Chen et al. reported they had prepared tanshinone Ⅱ_A (TSN)-GA solid lipid nanoparticles (GT-SLNs) and found a certain therapeutic effect on acne vulgaris [18]. However, the underlying mechanisms of the anti-acne effects of GA remain unclear. 

Network pharmacology is a concept and method that combines systems biology, bioinformatics and pharmacology and has been widely used in exploring the systemic effects of traditional Chinese medicine or compounds [19]. Network pharmacology combined with proteomics analysis has inspired new skills to elucidate drug mechanisms of action and to predict disease therapeutic targets. In this study, we explored the anti-acne effect and possible therapeutic mechanism of GA. First, we predicted the key targets and possible mechanisms of GA against acne vulgaris through network pharmacological analysis. Then, we adopted molecular docking and pharmacological experiments in vitro and in vivo as well as proteomics analysis to verify the anti-acne effects and molecular mechanisms of AG. This study offers a new perspective to investigate the therapeutic mechanism of AG in the treatment of acne vulgaris, which will give effective guidance for the development of AG in clinical applications. 

## 2. Results

### 2.1. Network Pharmacology Analysis of GA against Acne Vulgaris

**Figure 1:**
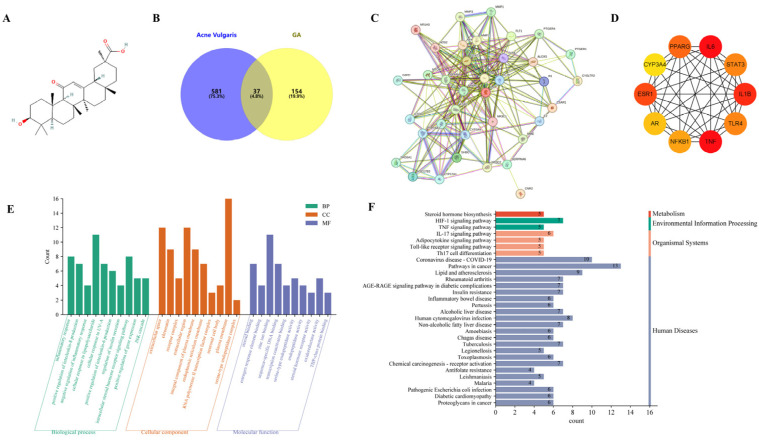
Network pharmacology analysis of GA and acne vulgaris. (**A**) Simplified molecular input line entry system (SMILES) for GA. (**B**) Venn diagram of target interaction between GA and acne vulgaris. (**C**) Interaction network of 37 targets of GA against acne vulgaris. (**D**) Top 10 targets by degree ranking. (**E**) GO analysis of intersecting genes. (**F**) Top 30 pathway terms presented by enriching the intersected targets in KEGG.

We first identified 37 targets through the intersection potential between pharmacological targets of GA and acne vulgaris-related targets (Figure 1B). As shown in Figure 1C, the PPI network was constructed by the STRING platform to show 37 nodes and 356 interactions, with an average node degree of 17. The results of the PPI were imported into Cytoscape to show the top 10 hub targets with the highest degree. The results suggest that tumor necrosis factor (TNF), interleukin-1β (IL-1β), interleukin-6 (IL-6), Estrogen Receptor alpha (ESR1), peroxisome proliferator-activated receptor gamma (PPAR-γ), Nuclear factor kappa B subunit 1 (NFκB1), signal transducer and activator of transcription 3 (STAT3) and Toll-like receptor 4 (TLR4) may be the most critical targets of Glycyrrhetinic acid against acne vulgaris (Figure 1D).

Next, the Gene Ontology (GO) analysis enriched 199 results in biological processes (BPs), 17 results in cellular component (CC) and 39 results in molecular function (MF) (*p* < 0.05), and the first 10 items were visualized (Figure 1E). Interestingly, BPs are principally implicated in the regulation of interleukin-8 production, inflammatory response and cellular response to lipopolysaccharide. Steroid binding and estrogen response element binding were highlighted as the most important MFs, while extracellular space, chromatin, receptor complexes and extracellular region were identified as the most enriched terms for CC. Kyoto Encyclopedia of Genes and Genomes (KEGG) pathway enrichment analysis identified 72 pathways, and the top 30 pathways were visualized in Figure 1F. Surprisingly, the intersecting targets were significantly associated with steroid hormone biosynthesis and adipocytokine and TNF and IL-17 signaling pathways, suggesting that GA may have a regulatory effect on sebaceous lipogenesis and inflammatory response. 

### 2.2. Molecular Docking

**Figure 2:**
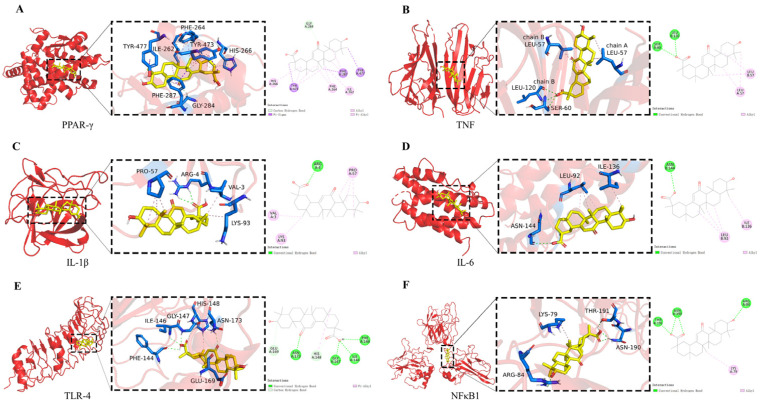
Docking pattern between GA and the core target proteins. The ligand–protein binding interactions between GA and PPAR-γ (**A**), TNF (**B**), IL-1β (**C**), IL-6 (**D**), TLR-4 (**E**) and NFκB1 (**F**), respectively.

PPAR-γ, TNF, IL-1β, IL-6, TLR and NFκB1 were identified as potential core targets of GA in the regulation of acne vulgaris. The AutoDock docking tool was used for molecular docking between GA and six potential core targets. The binding results are shown in Figure 2 and Table 1. All the binding energies are negative, indicating that GA and protein can bind spontaneously, and the lower the binding energy, the easier GA will bind to the protein. PPAR-γ may be the core target of GA against acne, with the strongest binding effect, because the binding energy of PPAR-γ to GA was −11.0 kcal/mol.

### 2.3. GA Inhibits Dex-Induced Lipogenesis and Inflammation in Human SZ95 Sebocytes

**Figure 3:**
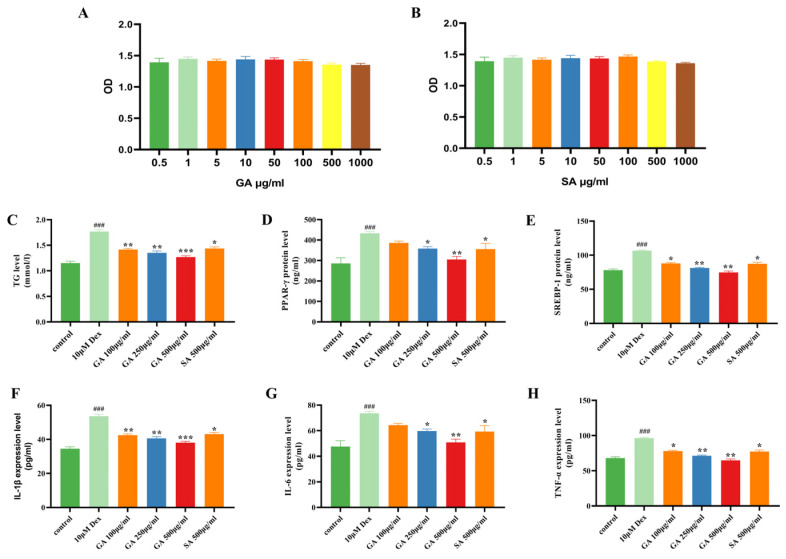
GA relieved Dexone (Dex)-induced inflammation and inhibited the levels of TG, PPAR-γ and SREBP-1 in human SZ95 sebocytes. (**A**,**B**) Effects of GA and SA at different concentrations (0–1000 μg/mL) on cell viability of SZ95 cells. (**C**–**E**) Protein levels of TG, PPAR-γ and SREBP-1 in SZ95 measured by ELISA. (**F**–**H**): Pro-inflammatory cytokines of TNF-α, IL-1β and IL-6 in SZ95 cells were determined by ELISA methods. Values are expressed as the means ± SD (n = 3), * *p* < 0.05, ** *p* < 0.01 and *** *p* < 0.001, compared with the Dex group. ### *p* < 0.001, compared with the control group.

The cell viability of SZ95 treated with different concentrations of GA (5, 10, 50, 100, 500, 1000 μg/mL) was first determined by MTT compared with salicylic acid (SA), an anti-inflammatory and keratolytic agent [20], which is used to remove follicular clogs and to reduce comedones [21]. As shown in Figure 3A,B, the GA and SA had no effect on cell viability in SZ95 cells in a concentration ranging from 5 to 1000 μg/mL.

Then, human SZ95 sebocytes were co-treated with Dex or DMSO and GA or SA for 48 h to determine the potential regulatory effects of GA on sebaceous lipids and inflammatory response. The evidence showed that dexamethasone (Dex) induced sebaceous lipid production, which could be regulated through the expression of the transcription factor sterol response element-binding protein 1 (SREBP-1) [22]. As shown in Figure 3C–H, Dex induction not only increased the levels of triglyceride (TG), SREBP-1 and PPAR-γ involved in fatty acid storage and glucose metabolism but also up-regulated the pro-inflammatory cytokine expression (TNF-α, IL-1β and IL-6). However, these changes were improved with GA treatment in a concentration-dependent manner. Moreover, GA had a stronger inhibition of the sebaceous lipogenesis and inflammatory response than SA at the same dose of 500 μg/mL in Dex-induced SZ95 cells.

### 2.4. Effect of GA on a Mouse Model of Acne Vulgaris

**Figure 4:**
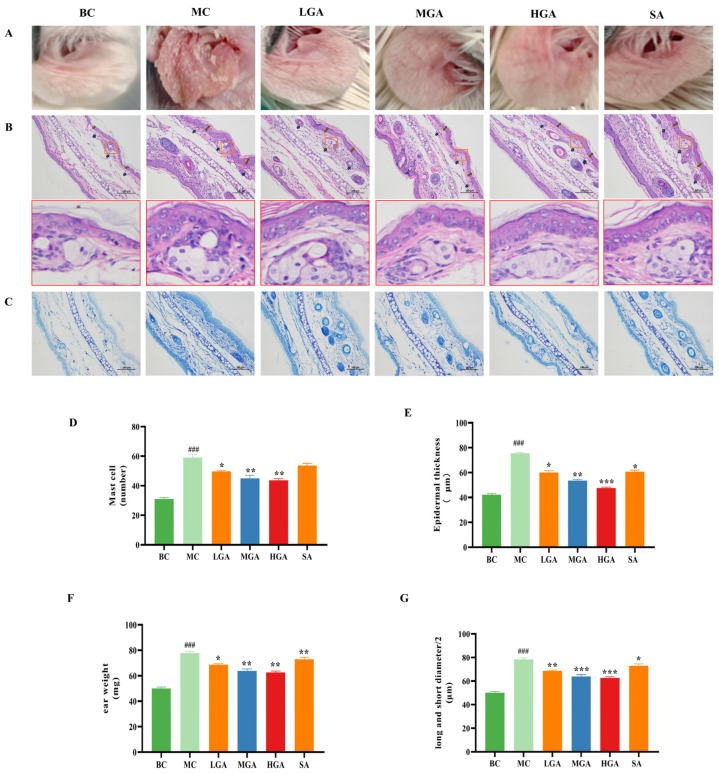
Effect of GA on a mouse model of acne vulgaris. Blank control group (BC), acne model group (MC), high-dose GA-treated group (HGA, 5 mg/mL), medium-dose GA-treated group (MGA, 2.5 mg/mL), low-dose GA-treated group (LGA, 1 mg/mL) and SA-treated group (SA, 5 mg/mL). (**A**) Photographs of mouse acne ear skin. (**B**) H&E staining of mouse ear skin (magnification ×200). The black arrow represents the SGs. The brown arrow represents the epidermal thickness. The red boxes represent enlarged and blocked sebaceous glands (magnification ×2000). (**C**,**D**) Number of mast cells in the toluidine blue-stained tissue sections. (**E**) Mouse ear epidermal thickness. (**F**) Mouse ear weight. (**G**) Half of the short and long diameters of SGs. All the values are the mean ± SD (n = 6), * *p* < 0.05, ** *p* < 0.01 and *** *p* < 0.001, compared with the MC group. ### *p* < 0.001, compared with the control group.

To explore the anti-lipid and anti-inflammatory effects of GA compared to those produced by SA, BALB/c mice, treated with oleic acid (OA) and then injected with *P. acnes*, were used as an in vivo model for acne vulgaris. We noted that the acne injection initiated an obvious inflammatory response in the ear skin of the model group (MC group). As shown in Figure 4A, ear edema, erythema and rough skin surface symptoms were clearly observed in the MC group, while these symptoms were clearly ameliorated in the GA-treated group. Then, a histopathological examination of the acne-like mouse ear skin lesions was also performed. As shown in Figure 4B,E, we found an increase in the epithelial cells within the funnel-shaped part of the hair follicle structure and a thickened layer of the stratified squamous epithelium in the acne-like mouse ear skin lesions. Indeed, the thickness of the epidermal layer of the acne-like mouse ear skin lesions increased to 75.56 ± 2.35 μm, compared to that in the normal mice (42.23 ± 1.13 mm μm), whereas GA treatment decreased the *P. acnes*-induced epidermal thickness to 60.03 ± 1.89 (L), 53.53 ± 2.06 (M) and 47.51 ± 1.12 (H) μm, respectively. SA treatment reduced the epidermal thickness to 60.73 ± 0.89 μm at a dose similar to the high dose of GA. Meanwhile, the H&E staining results revealed that the *P. acnes* injection increased the number of infiltrated inflammatory cells, which is further confirmed by the staining with toluidine blue. As shown in Figure 4C,D, mast cells were markedly enhanced in the dermis layer of the ear skin of the *P. acnes*-induced BALB/c mice compared with those in the normal mice (BC group). On the contrary, GA treatment significantly suppressed the number of mast cells. In addition, the weight of the *P. acnes*-induced ear skin lesions on the BALB/c mice, which can indicate the signs of skin edema, was measured. As shown in Figure 4F, the *P. acnes*-induced ear skin lesions resulted in ear edema, compared with that in the BC group. On the contrary, the ear swelling was obviously ameliorated after treatment with GA, which showed a better effect than SA in inhibitory activity. These data showed that treatment with GA could efficiently ameliorate the acne symptoms including epidermal thickening and swelling of the skin, as well as inflammatory infiltration in *P. acnes*-induced BALB/c mice, which is possibly attributed to the recovery of skin damage caused by *P. acnes*. Moreover, it is worth noting that the SGs were enlarged and the skin’s hair follicles became blocked (Figure 4B,G) due to the hyperkeratinization of the hair follicles in *P. acnes*-induced BALB/c mice, compared with those in the normal mice. However, these alterations were ameliorated with the treatment of GA, especially for the high dose, displaying GA-treated skin tissues similar to those of the BC group in terms of the structure of the skin’s epidermal layer and the sebaceous glands, as well as the epidermal thickness of the entire ear.

### 2.5. GA Suppresses Sebogenesis and Pro-Inflammatory Cytokines in a Mouse Model of Acne Vulgaris

**Figure 5:**
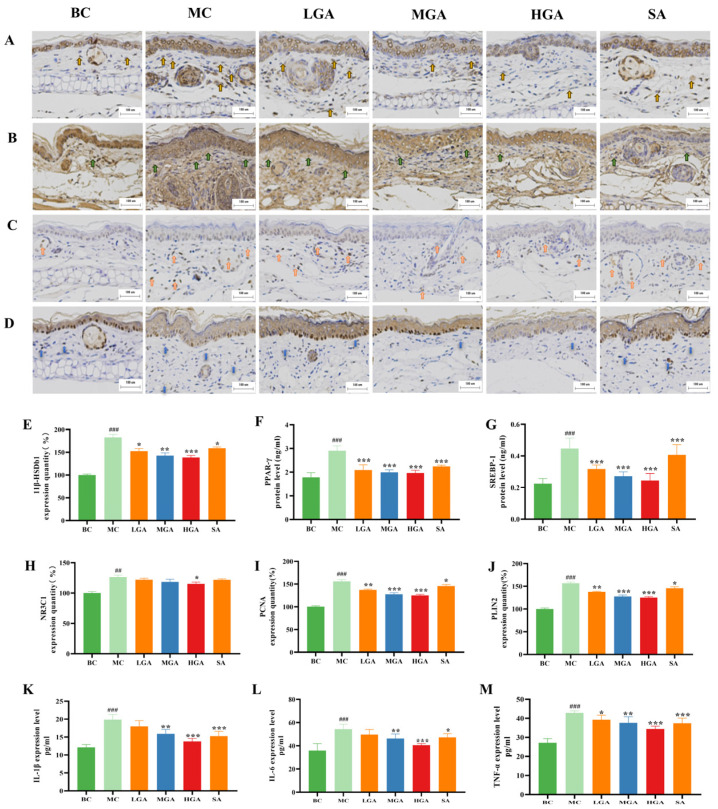
GA suppresses sebogenesis and pro-inflammatory cytokines. (**A**–**D**) Immunohistochemical labeling of 11β-HSDb1, PLIN2, NC3R1 and PCNA in the ear skin of mouse acne model (magnification 200×, scale bar = 100 μm); the yellow, green, orange and blue arrows indicate the abundance of 11β-HSDb1, PLIN2, NC3R1 and PCNA proteins. (**E**–**H**) 11β-HSDb1, PPAR-γ, SREBP-1 and NC3R1 expression levels of mouse ear skin tissues. (**I**,**J**) PLIN2 and PCNA expression levels of mouse ear skin measured by ELISA assay. (**K**–**M**) IL-1β, IL-6 and TNF-α expression of mouse ear skin measured by ELISA assay. All the values are the mean ± SD (n = 6), * *p* < 0.05, ** *p* < 0.01, *** *p* < 0.001, compared with the MC group. ## *p* < 0.05, ### *p* < 0.001, compared with the control group.

To uncover the possible molecular mechanisms of the anti-acne actions of GA, we first examined the role of GA on sebogenesis in a mouse model of acne vulgaris. The sebogenesis-related factors, including sterol regulatory element-binding protein 1 (SREBP-1), 11β-hydroxysteroid dehydrogenase (11β-HSD), peroxisome proliferator-activated receptor (PPAR)-γ and glucocorticoid receptor (GR), also named as NR3C1 (nuclear receptor subfamily 3, group C, member 1), in the *P. acnes*-induced acne-like mouse ear skin lesions were determined by immunohistochemical staining (Figure 5A,C) and ELISA assays (Figure 5E–H). We noted that the expression of PPAR-γ, SREBP-1, 11βHSD and NR3C1 in the acne-like mouse ear skin lesions were obviously higher than those observed in the normal mice, whereas GA significantly down-regulated the expression of these proteins.

Then, the proliferation of sebaceous gland cells and lipid droplet proteins such as perilipin (PLIN) 2 were also explored through immunohistochemical staining of the *P. acnes*-induced mouse ear skin lesions (Figure 5B,J). As shown in Figure 5I,J, the results demonstrated that the expression of PLIN 2 and PCNA was obviously higher than that noticed in the normal mice, whereas GA treatment decreased the expression levels of these proteins in a concentration-dependent manner. 

To investigate the anti-inflammatory effects of GA treatment in *P. acnes*-induced mouse ear skin, we analyzed pro-inflammatory cytokines such as TNFα, IL-1β and IL-6, which are well known to be up-regulated in acne skin lesions [23,24]. As shown in Figure 5K–M, a significant increase in inflammatory cytokine (IL-6, TNFα and IL-1β) release was observed in the ear skin lesions of the BALB/c mice induced by *P. acnes* (*p* < 0.001), whereas topical treatment of GA showed a concentration-dependent downregulation of cytokines and stronger inhibitory effects on cytokines than SA.

### 2.6. Differentially Expressed Proteins (DEPs) Analysis

**Figure 6:**
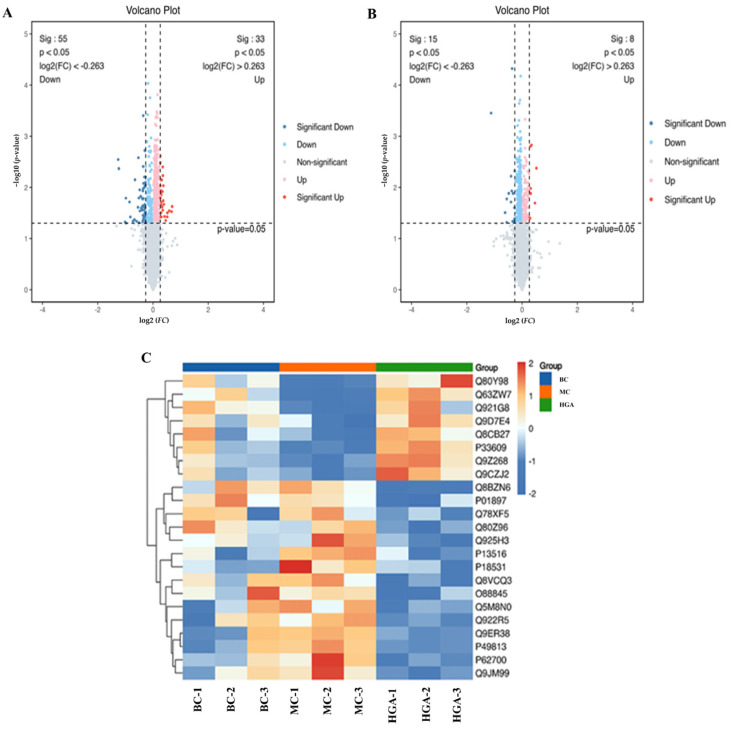
Proteomic analysis of skin (**A**) volcano plot of DEPs in BC group vs. MC group. The horizontal coordinate of the volcano map is log2 and the ordinate is −log10 (*p*-value). Blue and red dots indicate up-regulated and down-regulated proteins, respectively. Darker colors indicate more significant differences, and gray dots represent proteins with *p*-values ≥ 0.05. (**B**) Hierarchical clustering heat map of 23 DEPs between GA group and MC group. (**C**) The protein expression is clustered according to the amount of protein expression. The colors from red to blue indicate high expression levels to low expression levels in the DEPs.

A total of 6483 proteins were obtained through quantitative proteome analysis, of which 6348 proteins were quantitatively analyzed. The differentially expressed proteins (DEPs) are shown in Figure 6A,B by volcano plots. Of these, 33 up-regulated and 55 down-regulated proteins were screened in the acne model group (MC) versus the blank control group (BC) in accordance with the DEP standards of a fold change of over 1.2- or < 1/1.2-fold, as well as *p* < 0.05. In addition, there were a total of 23 DEPs in the GA-treated groups versus the acne model group (MC). Among them, 8 DEPs were up-regulated, and 15 DEPs were down-regulated. In addition, hierarchical clustering analysis of these DEPs shown in Figure 6C revealed the pathogenesis of acne was closely related to differential protein expression levels, including nuclear receptor-binding factor 2 (NRBF-2), cannabinoid receptor interacting protein 1a (CRIP1a) and acyl-CoA desaturase 1 (SCD-1), Phospholipase DDHD2 and Proteoglycan 4 (Prg4).

### 2.7. GO and KEGG Pathway Analysis

**Figure 7:**
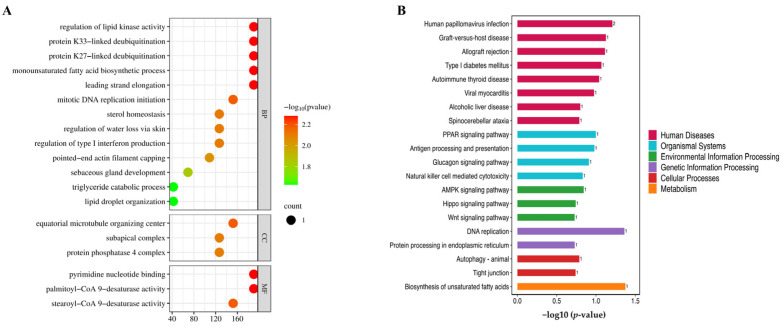
GO and KEGG enrichment analysis. (**A**) GO analysis of DEPs. The horizontal coordinates represent the enrichment score, and the vertical coordinates represent the top 20 terms information of BP/CC/MF, respectively. The color of the bubbles changes from blue to red. The redder the color, the smaller the *p* value and the more significant the enrichment. Larger bubbles contain more protein. (**B**) KEGG analysis of DEPs.

GO enrichment analysis of DEGs in GA/BC groups (Figure 7A) and BC/BC groups (Figure S1A,B) was first conducted against the GO database by using three independent ontologies, including molecular functions, cellular component categories and biological processes. The GO analysis demonstrated that the major enriched cellular components in the GA group were centriolar satellite, microtubule organizing, center nuclear envelope, chromatin and centrosome. DEPs in the GA group were associated with the regulation of molecular functions, such as metal ion binding, ATP binding, type 1 cannabinoid receptor binding, purine nucleotide binding and pyrimidine nucleotide binding. These DEPs participated in the biological processes of the immune response, tarsal gland development, lagging strand elongation, leading strand elongation and regulation of lipid kinase activity. It is worth noting that there was an obvious difference in GO enrichment among BC, MC and GA groups, which was associated with acne pathology. Based on KEGG pathway enrichment analysis, DEPs in the GA group were mainly the PPAR, AMPK and Wnt signaling pathways (Figure 7B). 

### 2.8. Validation of Proteins Identified by Proteomics Analyses

**Figure 8:**
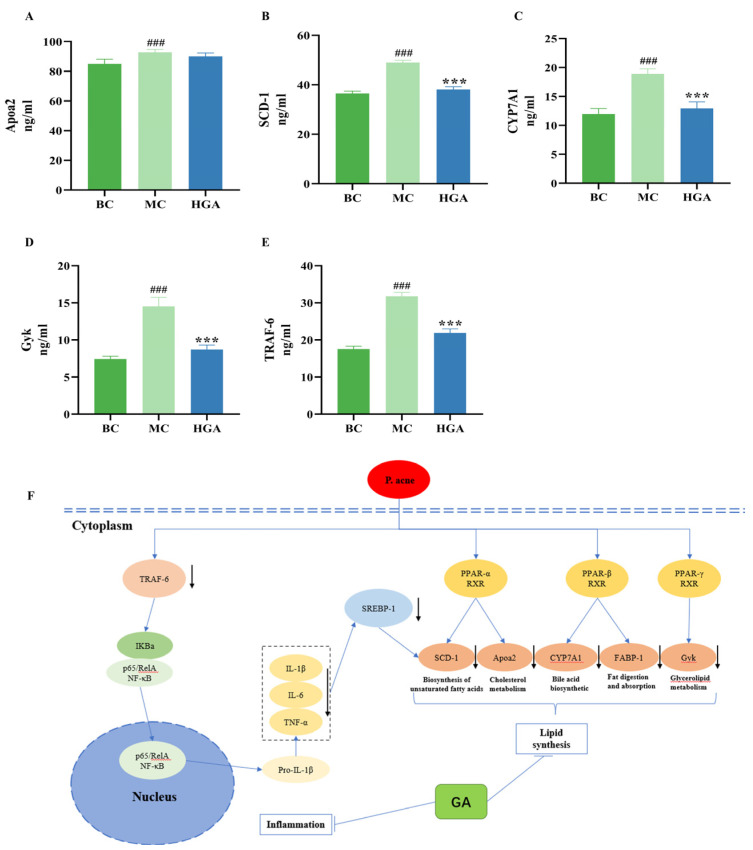
Validation of the candidate proteins. (**A**–**E**) Relative expression of Apoa2, SCD-1, CYP7A1, Gyk and TRAF-6. Values are shown as the means ± SD (n = 6), ### *p* < 0.001, compared with the BC group. *** *p* < 0.001, compared with the MC group. (**F**) Hypothesized mechanisms of GA in the treatment of acne.

Through the analysis of the above results, the proteins identified by proteomics analyses (Apoa2, SCD-1, CYP7A1, Cyk and TRAF-6), which play an important role in acne pathology and are altered in the intervention by GA treatment (Figure 8F), were further analyzed by ELISA. Gyk is a key enzyme in fatty acid esterification, and the activation of the PPAR pathway triggers an increase in Gyk expression, further promoting triglyceride synthesis. Apoa2, SCD-1, CYP7A1 and p-akt are downstream factors of the PPAR pathway. TRAF6 is a key mediator of NF-κB signaling pathways, thereby regulating downstream gene expression. As shown in Figure 8A–E, Apoa 2, SCD-1, CYP7A1, Gyk, and P-akt were significantly up-regulated in the MC group compared to the BC group (*p* < 0.05). Conversely, SCD-1, CYP7A1, Cyk and P-akt were significantly down-regulated in the GA group compared to the MC group. However, the expression of Apoa 2 in the GA group was not statistically different from that of the MC group.

## 3. Discussion

Increasing evidence has emerged that inflammation manifests throughout the development of acne lesions [25]. *P. acnes* is known to be an important pathogenic factor for the development of acne. Colonization of the pilosebaceous follicle by *P. acnes* stimulates the expression of IL-1β, TNF-α, IL-6 and IL-8 through activating Toll-like receptors (TLRs) and induces the expression of nitric oxide synthase (iNOS) and cyclooxygenase-2 (COX-2) in human sebocytes, thereby amplifying the inflammatory cascade and deteriorating acne vulgaris [26,27]. In this study, the network pharmacological and proteomic analyses were used to explore the mechanism of GA in treating acne vulgaris. A total of 37 potential targets were predicted by network pharmacology, among which TNF, IL-1B, IL-6, ESR1, PPARG, NFκB1, STAT3, and TLR4 may be the key targets of GA in the treatment of acne. Molecular docking showed that GA exhibited high affinities with TNF, IL-1β, IL-6, PPAR-γ, NFκB1, and TLR4. IL-1β is a potent inducer of pro-inflammatory cytokine production such as IL-6 and IL-8 in sebocytes (the sebaceous gland cells), which accumulate lipids and lipid droplets (LDs) and release sebum. IL-1β mRNA and IL-1β are abundant in inflammatory acne lesions [28]. IL-6 is one of the major acne lesion-associated cytokines and is predominant in the inflammatory lesions of acne vulgaris [29]. TNF-α is closely involved in mediating inflammatory and innate immune responses. The experimental results showed that there is an obvious up-regulation of TNF-α, IL-1β and IL-6 in Dex-induced SZ95 sebocytes and in the lesional skin of BALB/c mice induced by *P. acnes,* while GA obviously prevented the secretion of IL-1β, IL-6 and TNF-α in vitro et in vivo. 

Proliferating cell nuclear antigen (PCNA) was originally characterized as an antigen expressed in the cell nucleus during the DNA synthesis in the S-phase of the cell cycle. The PCNA was immunohistochemically stained in this study to investigate the proliferation of sebaceous gland cells. We noted that PCNA-positive cells significantly increased in *P. acnes*-induced mouse ear skin lesions, whereas GA treatment could obviously reduce the expression of PCNA in a dose-dependent way, suggesting that GA had an inhibitory activity on the proliferation of sebaceous gland cells. Indeed, the sebaceous enlargement was observed in *P. acnes*-induced acne-like ear skin lesions. However, we found that the size of SGs obviously decreased after GA treatment. LDs, also known as lipid bodies, are lipid-rich cellular organelles, which modulate the storage, transportation and metabolism of lipids and play a crucial role in the lipid homeostasis system. In adipocytes and sebocytes, the lipid droplet proteins, such as the perilipin (PLIN) family proteins segregate lipids by protecting lipid droplets from lipase action, dominate the levels of comedogenic free fatty acids and play important roles in lipid metabolism and lipid-related pathologies, including acne vulgaris. PLIN2, as a major member of the PLIN family proteins in sebocytes, is implicated in the accumulation of sebaceous lipids and the regulation of SG size. The experimental results demonstrated that PLIN2 was more intensely expressed in the acne-like ear skin lesions. However, GA treatment obviously reduced the expression level of PLIN2 in the *P. acnes*-induced acne-like ear skin lesions in a concentration-dependent manner. 

SREBP-1 is a major transcriptional factor, primarily responsible for the metabolism of fatty acids, which plays a key role in lipid synthesis [30,31]. PPAR-γ is recognized for its role in lipid metabolism and the storage of fatty acids, which stimulates adipogenesis and lipid uptake [32]. NR3C1 binds to cortisol and other glucocorticoids and increases the expression levels of anti-inflammatory mediators in the nucleus or suppresses the expression of pro-inflammatory mediators in the cytosol. 11βHSD catalyzes the conversion of inactive cortisone into active cortisone (glucocorticoid), which can be mediated by GR to stimulate sebocyte proliferation and promote lipid secretion from sebaceous glands [32]. Indeed, in this study, the experimental results revealed that the sebogenesis-related factors such as SREBP-1, PPAR-γ, NR3C1 and 11βHSD obviously increased, resulting in the lipid accumulation in *P. acnes*-induced acne-like ear skin lesions. However, GA treatment clearly inhibited the expression level of these factors in a concentration-dependent manner. Similar results were also found in Dex-induced SZ95 cells. 

Based on the TMT-labeled quantitative proteomic analysis, 23 DEPs before and after GA treatment were identified in the GA/BC groups. Among them, 15 DEPs were down-regulated, and 8 DEPs were up-regulated. The hierarchical clustering analysis of these DEPs demonstrated that the proteins in the acne-like ear skin in the GA group were closer to those in the BC group, whereas the proteins in the *P. acnes*-induced acne-like ear skin in the MC group were detached from those in the BC/GA groups. 

The KEGG pathway enrichment analysis showed GA treatment might involve multiple pathways including PPARs, biosynthesis of unsaturated fatty acids, AMPK, autophagy and Wnt, in which the DEPs, including acyl-CoA desaturase 1 (SCD-1), vang-like protein 1 (VANGL1), Nuclear receptor-binding factor 2 (NRBF2), Dual specificity phosphatase 14 (Dusp14), Alpha-1,6-mannosyl-glycoprotein 1-beta-N-acetylglucosaminyltransferase (Mgat1), Tenomodulin (Tnmd) and Vang-like protein 1 (Vangl1), were significantly altered (*p* < 0.05). These DEPs play a crucial role in the pathogenesis of acne vulgaris. We found that Scd1, Nrbf2, Mgat1 and Tnmd were significantly expressed in the PPAR signaling pathway. PPARs (PPARα, -β/δ and -γ) are a group of nuclear receptor proteins that are expressed in keratinocytes, sebocytes and hair appendages and function as transcription factors regulating the expression of lipogenic genes, the innate immune system and inflammatory responses. Apart from its anti-inflammatory properties, PPAR activation promotes sebocyte lipogenesis and increases lipid accumulation in human sebocytes [33]. Nrbf2 interacts with PPAR ligands and regulates PPAR-related lipid synthesis [34]. Mgat1 has a key role in hepatic TG accumulation. The inhibition of MGAT1 expression levels efficiently reduces lipid accumulation through the overexpression of PPARγ in HepG2 cells [35]. Tnmd, a new family of type II transmembrane glycoproteins, can accelerate adipogenic differentiation and significantly increase the mRNA level of PPAR-γ [36]. SCD-1, known as a key enzyme in the biosynthesis of unsaturated fatty acids pathway, is involved in the formation of lipid droplets and the accumulation of triglycerides [37]. In our analysis, Nrbf2 in the MC group is down-regulated, while Scd1, Mgat1 and Tnmd are up-regulated, compared with the BC group. Conversely, after treatment with GA, the Nrbf2 is up-regulated, while the Scd1, Mgat1 and Tnmd are down-regulated. This is consistent with our prediction that core genes are mainly involved in signaling pathways related to lipid synthesis and inflammation. 

## 4. Materials and Methods

### 4.1. Materials

GA (Purity > 99.2%) was obtained from Xinjiang Technical Institute of Physics and Chemistry, Chinese Academy of Sciences (Xinjiang, China). Salicylic acid (SA) was purchased from Aladdin (Shanghai, China). Oleic acid was provided by Macklin Biochemical (Shanghai, China). FBS, SP, PBS and trypsin-EDTA (0.25%) were bought from Gibco (Norristown, PA, USA). The BCA protein assay kit and MTT were obtained from Beyotime Biotechnology (Shanghai, China). The *Propionibacterium acnes* solution was bought from BeNa Biotechnology (Beijing, China). TG, SREBP-1, PPAR-γ, IL-6, IL-1β and TNF-α ELISA kits were bought from JiangLai Biotechnology (Shanghai, China). Anti-11β-HSDb1, anti-PCNA, anti-PLIN2 and anti-NR3C1 antibodies were provided by Servicebio Biotechnology (Wuhan, China). Human sebaceous gland cells (SZ95) were obtained from Dr. Zouboulis CC.

### 4.2. Collecting Potential Targets of GA

Potential targets of GA were obtained from the SwissTargetPrediction (http://www.swisstargetprediction.ch, accessed on 22 June 2023), Super-PRED (https://prediction.charite.de/subpages/target_prediction.php, accessed on 22 June 2023) and Similarity ensemble approach (https://sea.bkslab.org/, accessed on 22 June 2023) [38,39]. Then, the targets collected by the three platforms were summarized in Microsoft Excel, and duplicate values were removed. Finally, the Uniprot ID corresponding to the collected target names was imported into the UniProtKB (https://www.uniprot.org/, accessed on 22 June 2023) protein database to acquire the gene names of the standardized total proteins.

### 4.3. PPI Analysis

Venny 2.1.0 online tools (https://bioinfogp.cnb.csic.es/tools/venny/index.html, accessed on 22 June 2023) were used to analyze the intersections of acne vulgaris-related targets and GA targets. Potential targets were imported into STRING (www.string-db.org/, accessed on 22 June 2023) and analyzed to provide a protein–protein interaction (PPI) network diagram [40]. The degrees between targets in the PPI were analyzed using Cytoscape software (version 3.9.0) to give 10 hub targets with the highest degrees.

### 4.4. Molecular Docking

Six potential core protein targets were selected for docking with GA. The GA and proteins were pre-processed with AutoDockTools 1.5.7, and then molecular docking was performed with AutoDock Vina (version 1.2.0) [41]. The affinity fraction represents the binding energy between the GA and the protein. The smaller the affinity fraction, the stronger the interaction between the GA and the protein. 

### 4.5. Cell Culture

SZ95 cells were grown in Sebomed basal medium with 10% fetal bovine serum, 1% penicillin/streptomycin and human epidermal growth factor (5 ng/mL) and incubated at 37 °C in a humidified atmosphere with 5% CO_2_. 

### 4.6. Cell Viability Essay

Cell viability was assessed using the MTT assay. Briefly, SZ95 cells were transferred to 96-well plates (5 × 10^3^ cells/well), GA or SA in PBS was added at final concentrations of 0.5, 1.5, 10, 50, 100, 500 and 1000 μg/mL and allowed to incubate for 24 h. Then, MTT solution was added to each of the wells and maintained for 4 h. Finally, the MTT was removed and DMSO was added to solubilize the formazan precipitate. The optical density (OD) in each well was measured at 570 nm with a microplate reader (Bio-Tek, Shoreline, WA, USA).

### 4.7. Enzyme-Linked Immunosorbent Assay (ELISA)

SZ95 sebocytes were seeded in a 96-well flat-bottomed plate (5 × 10^3^ cells/well) and incubated at 37 °C for 24 h in an incubator containing 5% CO_2_. After washing with phosphate-buffered saline (PBS), Different concentrations (100, 250 and 500 μg/mL) of GA or SA (500 μg/mL) were added and followed by incubation for 24 h. Following lysis with RIPA lysis buffer and PMSF, the lysates were centrifuged at 12,000× *g* at 4 °C for 10 min to give the supernatant. The mouse ear skin tissues were homogenized with RIPA lysate buffer for 5 min using a tissue grinder and then centrifuged at 4000× *g* at 4 °C for 10 min to produce the supernatant. 

The levels of TG, SREBP-1, PPAR-γ, IL-1, IL-6 and TNF-α were determined using commercial ELISA kits, according to the manufacturers protocol, respectively. The OD was measured at 450–550 nm using a microplate reader (Bio-Tek Infinite M200 Pro, Shoreline, WA, USA). The quantitative determination of total protein in the samples was performed using the BCA protein assay method.

### 4.8. Mice Treatments

BALB/c mice (18–22 g, 7–8 weeks, male) were obtained from Guangzhou Laboratory Animal Center (Guangzhou, China) and kept under pathogen-free conditions at 23 ± 2 °C, 50 ± 10% humidity, with filtered air and 12 h/12 h light:dark cycles and fed a regular diet and sterilized water. The mice were randomly divided into five groups (n = 6): blank control group (BC), acne model group (MC), high-dose GA-treated group (5 mg/mL, HGA), medium-dose GA-treated group (2.5 mg/mL, MGA), low-dose GA-treated group (1 mg/mL, LGA) and SA-treated group (5 mg/mL, SA). Each mouse’s ear skin was evenly smeared with 50 μL of 80% oleic acid (OA) and injected with 50 μL of a *P. acnes* solution (1 × 107 colony-forming units/μL) once per day for 14 days, except for the BC group. After 4 h, 100 μL of GA or SA solution was applied to the ear skin once daily for 14 days. All the mice were euthanized on day 15. The ear skin was removed and kept at −80 °C for further analysis.

### 4.9. Histopathological Examination

Paraffin-embedded tissue of the mouse ear skin was prepared as described in previous studies and then subjected to H&E staining [42]. The H&E stained tissue slides were analyzed by an optical microscope (BX53, Olympus, Tokyo, Japan). 

### 4.10. Immunohistochemistry

The paraffin sections of mouse ear skin were placed in a citrate buffer (0.01 M) at 121 °C for 20 min and 1% BSA for 20 min. The sections were treated with primary antibodies (anti-HSD11b1, anti-PLIN2, anti-NC3R1 and anti-PNCA) for 16 h at 4 °C and secondary antibody (goat anti-rabbit IgG-HRP) for 1 h at 4 °C. All the slides were photographed under an optical microscope. The positive chromogenic optical density values were quantitatively determined by IPP 6.0 software.

### 4.11. Establishment of a Target Database for Acne Vulgaris

The target sites of acne vulgaris were collected by typing “Acne Vulgaris” into the search box on the GeneCards website (www.genecards.org, accessed on 15 October 2023), and the results were exported.

### 4.12. Protein Digestion and TMT Labeling

Diphtheria–tetanus toxoid (DTT, 5 mM) was added to the supernatants from the same mouse ear skin tissue, obtained as above, and the samples were incubated at 55 °C for 30 min. Then, 10 mM iodoacetamide was added and protected from light at room temperature for 15 min. The protein extracts were precipitated with six times the volume of cold acetone, maintained at −20 °C overnight and followed by centrifugation (8000 rpm) at 4 °C for 10 min. The collected protein was dissolved in 100 μL of 200 mM triethylammonium bicarbonate (TEAB) with 1 mg/mL Trypsin-TPCK (1/50 ratio) and kept overnight at 37 °C. Each freeze-dried sample was labeled with a TMT reagent, according to the manufacturer’s protocol.

The TMT-labeled peptide separation was performed by reversed-phase (RP) chromatography with an Agilent 1100 HPLC. The peptides were eluted using Phase A (CH_3_CN-H_2_O, 2:98, *v*/*v*) and Phase B (CH_3_CN-H_2_O, 90:10, *v*/*v*) on a narrow diameter Agilent Zorbax-C18 column (2.1 × 150 mm, 5 μm) at a flow rate of 300 μL/min under gradient conditions: 0–8 min, 98% Phase A; 8–8.01 min, 98–95% Phase A; 8.01–48 min, 95–75% Phase A; 48–60 min, 75–60% Phase A; 60–60.01 min, 60–10% Phase A; and 60.01–70 min, 10% Phase A. The samples collected were dried and frozen for mass spectrometry.

### 4.13. LC-MS/MS Analysis

The samples were loaded onto a narrow diameter Acclaim PepMap RSLC RP-C18 column (75 μm × 50 cm, 3 μm), which was coupled to a QExactive system of mass spectrometry. The peptide sample was analyzed using Phase A (CH_3_CN-H_2_O, 99.9–0.1, *v*/*v*) and Phase B (CH_3_CN-H_2_O-HCO_2_H, 80–19.9–0.1, *v*/*v*/*v*) at a flow rate of 300 μL/min under a gradient of 2–28% Phase B for 0~50 min; 28–42% Phase B for 50~60 min; 42–90% Phase B for 60~65 min; and 90% Phase B for 65~75 min. The MS scans were set to the full scanning charge–mass ratio m/z range of 350–1500 at the resolution of 60,000 with the automatic gain control value of 3 × 10^6^ and carried out on 20 of the highest peaks. All the MS/MS maps were obtained through high-energy collision cracking in the data-dependent positive ion mode, with collision energy at 32 eV, the maximum ion injection time (80 ms), the dynamic exclusion time (30 s) and the automatic gain control value of 2 × 10^5^ at a resolution of 45,000. 

### 4.14. Proteomic Bioinformatic Analysis

The protein ID obtained by LCMS was converted into the UniProt ID. Proteins with Score Sequest HT > 0, unique peptide ≥ 1 and no null expression values were screened from the original data retrieved from the UniProt database (http://ebi.ac.uk/GOA/, accessed on 15 October 2023). The trusted proteins were acquired after median normalization and log2 logarithmic conversion. On the basis of the trusted proteins, two criteria were chosen to calculate the difference between the samples. Fold change (log2 (fold change) = mean of the experimental group—mean of the control group) was used to evaluate the multiples of expression change between the samples. The *p*-value from the *t*-test shows the significance of the differences between the samples. The proteins meeting the criteria of fold change ≥ 1.2 or fold change ≤ 1/1.2 and *p*-value < 0.05 were set as DEPs (Tables S1 and S2). In the same way, proteins were set as trusted proteins if fold change ≥ 1.2 or fold change ≤ 1/1.2 (Tables S3 and S4). Then, GO annotations of the DEPs were accomplished with a community-based bioinformatics database (http://www.geneontology.org, accessed on 15 October 2023). KEGG pathway enrichment was performed with the online KEGG database (http://www.genome.jp/kegg/, accessed on 15 October 2023) [43].

### 4.15. Statistical Method

All the data were calculated using GraphPad Prism 7.0 and shown in the form of the mean ± standard deviation (SD). The differences among the group means were compared using an ANOVA test followed by the LSD multiple comparisons test. A *p*-value of less than 0.05 was determined to be statistically significant.

### 4.16. Ethics Statement

The ethical assessments related to the use of animal studies were approved by the Institutional Animal Care and Use Committee (IACUC), Center for Animal Experiments, Guangdong University of Technology (Guangzhou, China).

## 5. Conclusions

In conclusion, we predicted 37 potential GA targets, based on the network pharmacological and the TMT-labeled quantitative proteomic analysis. Among them, TNF, IL1B, IL6, ESR1, PPARG, NFKB1, STAT3 and TLR4 may be the key targets for GA in the treatment of acne vulgaris, which are further verified by molecular docking. The GO enrichment and KEGG pathway analyses revealed that the anti-acne targets of GA may regulate multiple pathways, including PPAR, the biosynthesis of unsaturated fatty acids, AMPK, autophagy and wnt. The experimental results confirmed that GA inhibited lipid synthesis in vitro and in vivo, improved the symptoms of acne vulgaris, prevented mast cell infiltration and decreased the level of pro-inflammatory cytokines, including TNF-α, IL-1β and IL-6. Furthermore, GA had an inhibitory activity on the proliferation of sebaceous gland cells, resulting in the reduction of sebaceous gland size. These results suggest the anti-acne mechanism of GA may be through the regulation of sebogenesis and inflammatory responses. However, further exploration will be required for the development of GA as a promising new drug for patients with acne vulgaris. 

## Figures and Tables

**Table 1:** Molecular docking of GA and core target proteins.

Gene Name	PDB ID/ Uniprot ID	Interactions	Cohesive Energy kcal/mol
PPAR-γ	6C5Q	Pi-Alkyl, Pi-Sigma	−11.0
TNF	2AZ5	Conventional hydrogen bond	−9.2
IL-1β	4DEP	Conventional hydrogen bond	−7.4
IL-6	1P9M	Conventional hydrogen bond	−8.0
TLR4	2Z62	Conventional hydrogen bond, Pi-Alkyl	−8.0
NFκB1	1VKV	Conventional hydrogen bond	−7.2

## Data Availability

The mass spectrometry proteomics data were deposited to the ProteomeXchange Consortium (http://proteomecentral.proteomexchange.org, accessed on 15 October 2023) via the iProX partner repository, with the dataset identifier PXD048028.

## Supplementary Materials

The following supporting information can be downloaded at: https://www.mdpi.com/article/10.3390/molecules29102345/s1, Figure S1: Gene Ontology annotation of DEPs between MC and BC; Table S1: Differential proteins between MC and BC; Table S2: Differential proteins between GA and MC; Table S3: Trusted proteins in skin between GA and MC; Table S4: Trusted proteins in skin between MC and BC.
