# Supplementary material for: Efficacy of Glycyrrhetinic Acid in the Treatment of Acne Vulgaris Based on Network Pharmacology and Experimental Validation
Source: Molecules. 2024 May 16;29(10):2345. doi: 10.3390/molecules29102345 (PMC11123902; doi:10.3390/molecules29102345)
Supplement: Supplementary file 1 [file molecules-29-02345-s001.zip › molecules-2956919-supplementary.pdf]

Efficacy of Glycyrrhetinic acid in the treatment of Acne Vulgaris through the regulation of sebogenesis and inflammatory responses

Lingna Xie, Congwei Ma, Xinyu Li, Huixiong Chen, Ping Han, Li Lin, Wei qiang Huang, Menglu Xu, Hailiang Lu, Zhiyun Du

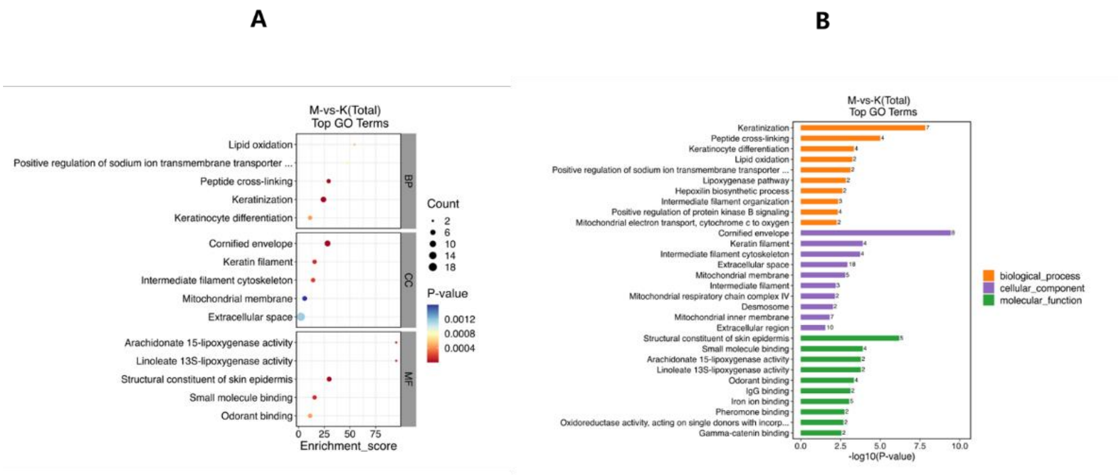

Fig. S1. Gene ontology annotation of DEPs between MC and BC. (A) GO analysis of DEP proteins. (B) The number of differentially expressed genes of GO.

Table S1: Differential proteins between MC and BC (Fold change ⩾ 1.2 or Fold change ⩽ 1/1.2 as well as p-value < 0.05)

protein name	protein definition	FoldChange	p-value	regulation
Sprr2d	Small proline-rich protein 2D	0.41770414	0.002850715	Down
Il36a	Interleukin-36 alpha	0.42486115	0.004289882	Down
Mup1	Major urinary protein 1	0.4964258	0.04731588	Down
Sprr1b	Cornifin-B	0.50667879	0.016256844	Down
Cdc42ep5	Cdc42 effector protein 5	0.5096846	0.049947698	Down
Alox8	Polyunsaturated fatty acid lipoxygenase ALOX8	0.55524254	0.019257919	Down
Mup17	Major urinary protein 17	0.56730769	0.037934468	Down
Chi3l1	Chitinase-3-like protein 1	0.60526829	0.045422791	Down
S100a8	Protein S100-A8	0.62890325	0.042789727	Down

Defb6	Beta-defensin 6	0.63719786	0.007092324	Down
Stfa3	Stefin-3	0.66278522	0.045800936	Down
Prss27	Serine protease 27	0.68656268	0.024418437	Down
Ccdc61	Centrosomal protein CCDC61	0.69416795	0.002619201	Down
Znf865	Zinc finger protein 865	0.70172451	0.016135879	Down
Glrx	Glutaredoxin-1	0.70397202	0.048927654	Down
Snrpc	U1 small nuclear ribonucleoprotein C	0.71672256	0.011183278	Down
Tmem14c	Transmembrane protein 14C	0.72053646	0.028858171	Down
Galk2	N-acetylgalactosamine kinase	0.73535018	0.014824575	Down
Mtnd1	NADH-ubiquinone oxidoreductase chain 1	0.74472516	0.025242296	Down
Casp14	Caspase-14	0.76281406	0.027881458	Down
Cox7a2	Cytochrome c oxidase subunit 7A2, mitochondrial	0.76351584	0.01637505	Down
Mt-Cyb	Cytochrome b	0.76620021	0.027039748	Down
Mug1	Murinoglobulin-1	0.7688958	0.049087931	Down
Fcgr3	Low affinity immunoglobulin gamma Fc region receptor III	0.76894021	0.016767048	Down
Hmgn3	High mobility group nucleosome-binding domain-containing protein 3	0.77173815	0.022998779	Down
Mpo	Myeloperoxidase	0.77553296	0.011729504	Down
Rpl35	60S ribosomal protein L35	0.77612271	0.044201633	Down
Serpina3k	Serine protease inhibitor A3K	0.77873804	0.035114983	Down
Cox6a1	Cytochrome c oxidase subunit 6A1, mitochondrial	0.78057906	0.01065072	Down
Ebp	3-beta-hydroxysteroid-Delta(8),Delta(7)-isomerase	0.78089294	0.003911956	Down
Smpdl3a	Acid sphingomyelinase-like phosphodiesterase 3a	0.78339582	0.000396936	Down
Atr	Serine/threonine-protein kinase ATR	0.78360038	0.023469103	Down
Gk	Glycerol kinase	0.78804322	0.016573551	Down
Tmem41b	Transmembrane protein 41B	0.78882107	0.008307636	Down
Slc37a2	Glucose-6-phosphate exchanger SLC37A2	0.79054345	0.03976601	Down
Sv2c	Synaptic vesicle glycoprotein 2C	0.80023927	0.0232147	Down
Ddhd2	Phospholipase DDHD2	0.80543124	0.027418713	Down
Mrpl12	39S ribosomal protein L12, mitochondrial	0.80621321	0.009992413	Down
Rprd2	Regulation of nuclear pre-mRNA domain-containing protein 2	0.80656349	0.0062639	Down
Arhgef18	Rho guanine nucleotide exchange	0.8131368	0.021631537	Down

	factor 18			
Spsb2	SPRY domain-containing SOCS box protein 2	0.81361603	0.001897452	Down
Ptma	Prothymosin alpha	0.81508363	0.031840756	Down
Ivl	Involucrin	0.81799277	0.009400323	Down
Ift140	Intraflagellar transport protein 140 homolog	0.81956295	0.036490498	Down
Nrm	Nurim	0.81961029	0.001916618	Down
Sprr1a	Cornifin-A	0.82086488	0.038577487	Down
Trmt44	Probable tRNA (uracil-O(2)-)-methyltransferase	0.82297281	0.025068847	Down
Dynlrb1	Dynein light chain roadblock-type 1	0.82379275	0.042710357	Down
Dcp2	m7GpppN-mRNA hydrolase	0.82580897	0.030897405	Down
Fam162a	Protein FAM162A	0.82662601	0.029528111	Down
Tubgcp2	Gamma-tubulin complex component 2	0.82791429	0.007986618	Down
Bnc2	Zinc finger protein basonuclin-2	0.82829313	0.046541406	Down
Znf706	Zinc finger protein 706	0.82971245	0.006207667	Down
Rasip1	Ras-interacting protein 1	0.83138003	0.009050863	Down
Atp6v0c	V-type proton ATPase 16 kDa proteolipid subunit c	0.83209876	0.049371639	Down
Dst	Dystonin	1.20161384	0.003310097	Up
Bpifb9a	Vomeromodulin	1.20389814	0.009260888	Up
Krt80	Keratin, type II cytoskeletal 80	1.20569918	0.016043604	Up
Mamdc2	MAM domain-containing protein 2	1.20714864	0.002461499	Up
Cyp2b10	Cytochrome P450 2B10	1.21196105	0.031246535	Up
Dennd10	DENN domain-containing protein 10	1.21447265	0.037845194	Up
Dsg1a	Desmoglein-1-alpha	1.21919875	0.01942447	Up
Cyp4b1	Cytochrome P450 4B1	1.21991729	0.007083344	Up
Flg2	Filaggrin-2	1.22147133	0.021134433	Up
Kprp	Keratinocyte proline-rich protein	1.22245667	0.013687904	Up
Mt4	Metallothionein-4	1.22380469	0.031357549	Up
Krt84	Keratin, type II cuticular Hb4	1.22509475	0.019164254	Up
Dsc3	Desmocollin-3	1.22532971	0.011617008	Up
Epha4	Ephrin type-A receptor 4	1.22897026	0.012255972	Up
Cnrip1	CB1 cannabinoid receptor-interacting protein 1	1.20969766	0.031644409	Up
Pip	Prolactin-inducible protein homolog	1.23475944	0.037781913	Up
Fhip1a	FHF complex subunit HOOK interacting protein 1A	1.24633624	0.007121315	Up
Obp1b	Odorant-binding protein 1b	1.25306391	0.02378033	Up
Nrbf2	Nuclear receptor-binding factor 2	1.20060491	0.041177047	Up

Bbox1	Gamma-butyrobetaine dioxygenase	1.26819799	0.012070902	Up
Tnnt1	Troponin T, slow skeletal muscle	1.27788676	0.00398389	Up
Npl	N-acetylneuraminate lyase	1.27832972	0.027811521	Up
Krt24	Keratin, type I cytoskeletal 24	1.28236474	0.005893034	Up
Alox12e	Polyunsaturated fatty acid (12S)/(13S)-lipoxygenase, epidermal-type	1.29315332	0.018297865	Up
F3	Tissue factor	1.2986056	0.009238786	Up
Cds2	Phosphatidate cytidylyltransferase 2	1.31272677	0.02149196	Up
Scd1	Acyl-CoA desaturase 1	1.17949284	0.020399987	Up
Plxna4	Plexin-A4	1.37611083	0.044238202	Up
Scgb2b24	Secretoglobin family 2B member 24	1.42868051	0.037693634	Up
Krt2	Keratin, type II cytoskeletal 2 epidermal	1.43651527	0.031367576	Up
Ighv3-6	Ig heavy chain V region 3-6	1.50429759	0.02869161	Up
Obp1a	Odorant-binding protein 1a	1.59567481	0.029756899	Up
Scgb2b2	Secretoglobin family 2B member 2	1.61772906	0.023615293	Up

Table S2. Differential proteins between GA and MC (Fold change ≥ 1.2 or Fold change ≤ 1/1.2 as well as p-value < 0.05)

protein name	protein definition	FoldChange	p-value	regulation
Tmod1	Tropomodulin-1	0.460078943	0.0003524	Down
Ighv3-6	Ig heavy chain V region 3-6 OS=Mus musculus	0.653725404	0.0310983	Down
Krtap6-5	Keratin-associated protein 6-5 OS=Mus musculus	0.680624033	0.0482403	Down
Ypel5	Protein yippee-like 5	0.681201413	0.0168121	Down
Nrbf2	Nuclear receptor-binding factor 2	0.71613658	0.0130472	Down
Prg4	Proteoglycan 4	0.757676199	0.0192882	Down
Dock10	Dedicator of cytokinesis protein 10	0.758399543	0.006042	Down
Ppp4r3b	Serine/threonine-protein phosphatase 4 regulatory subunit 3B	0.772269696	0.0117442	Down
Tor3a	Torsin-3A	0.779096173	4.743E-05	Down
Vangl1	Vang-like protein 1	0.78905075	0.045815	Down
Akap10	A-kinase anchor protein 10, mitochondrial	0.797973827	0.0131154	Down
Ostc	Oligosaccharyltransferase complex subunit OSTC	0.819805363	0.0460532	Down
Cnrip1	CB1 cannabinoid	0.826641291	0.0249413	Down

	receptor-interacting protein 1			
H2-L	H-2 class I histocompatibility antigen, L-D alpha chain	0.829329127	0.041222	Down
Scd1	Acyl-CoA desaturase 1	0.830775168	0.0047461	Down
C19orf25	UPF0449 protein C19orf25 homolog	1.200512146	0.0392879	UP
Tubgcp2	Gamma-tubulin complex component 2	1.222329578	0.0401076	UP
Pola1	DNA polymerase alpha catalytic subunit	1.234999108	0.0015736	UP
Yod1	Ubiquitin thioesterase OTU1	1.240332282	0.0131235	UP
Hspa12b	Heat shock 70 kDa protein 12B	1.241189445	0.0104855	UP
Patj	InaD-like protein	1.266695713	0.0014815	UP
Ddhd2	Phospholipase DDHD2	1.382922204	0.0202587	UP
Rasal1	RasGAP-activating-like protein 1	1.43397844	0.004211	UP

Table S3. Proteins of Trusted proteins in skin between GA and MC (Fold change ⩾ 1.2 or Fold change ⩽ 1/1.2).

Protein Name	Gene Name	UniProt ID	LogFC	P.values
Tropomodulin-1	Tmod1	P49813	-1.12005	0.00035
Keratin, type II cytoskeletal 2 oral	Krt76	Q3UV17	-1.02514	0.13988
Paired amphipathic helix protein Sin3b	Sin3b	Q62141	-1.02060	0.11420
Transmembrane protease serine 13	Tmprss13	Q5U405	-0.93702	0.10124
Ig gamma-2A chain C region, membrane-bound form	Igh-1a	P01865	-0.92768	0.05181
Deubiquitinase OTUD6B	Otud6b	Q8K2H2	-0.77006	0.08904
Keratin-associated protein 6-2	Krtap6-2	O08884	-0.66701	0.10246
Keratin-associated protein 19-2	Krtap19-2	Q925I0	-0.66670	0.08790
Interferon-induced protein with tetratricopeptide repeats 1	Ifit1	Q64282	-0.63485	0.13829
Ig heavy chain V region 3-6	Ighv3-6	P18531	-0.61324	0.03110
Keratin, type I cuticular Ha4	Krt34	Q9D646	-0.59875	0.09919
Keratin-associated protein 19-4	Krtap19-4	Q925H7	-0.57011	0.09407
Keratin-associated protein 6-5	Krtap6-5	Q925H3	-0.55507	0.04824
Protein yippee-like 5	Ypel5	P62700	-0.55385	0.01681
Keratin-associated protein 3-1	Krtap3-1	A2A591	-0.54636	0.07607
Keratin-associated protein 19-3	Krtap19-3	Q925H6	-0.54106	0.08251
Keratin-associated protein 21-1	Krtap21-1	Q925H4	-0.52841	0.08906
Protein phosphatase PTC7 homolog	Pptc7	Q6NVE9	-0.52803	0.21209
Keratin, type II cuticular Hb6	Krt86	P97861	-0.50838	0.10053
DNA polymerase alpha catalytic subunit	Pola1	P33609	-0.48169	0.01305

Keratin-associated protein 19-5	Krtap19-5	O08632	-0.47794	0.09745
Keratin, type II cytoskeletal 1b	Krt77	Q6IFZ6	-0.44363	0.35087
Keratin, type II cuticular 87	Krt87	Q6IMF0	-0.43930	0.12388
Keratin, type II cuticular Hb1	Krt81	Q9ERE2	-0.43783	0.09640
Keratin, type I cuticular Ha3-I	Krt33a	Q8K0Y2	-0.41794	0.10489
Keratin, type I cuticular Ha3-II	Krt33b	Q61897	-0.40698	0.10389
Proteoglycan 4	Prg4	Q9JM99	-0.40035	0.01929
Dedicator of cytokinesis protein 10	Dock10	Q8BZN6	-0.39897	0.00604
Dual specificity protein phosphatase 14	Dusp14	Q9JLY7	-0.38406	0.06769
Serine/threonine-protein phosphatase 4 regulatory subunit 3B OS=Mus musculus OX=10090 GN=Ppp4r3b PE=1 SV=2	Ppp4r3b	Q922R5	-0.37282	0.01174
Keratin-associated protein 14	Krtap14	O08640	-0.37194	0.26308
Alpha-1,6-mannosyl-glycoprotein 1-beta-N-acetylglucosaminyltransferase	Mgat1	Q921V5	-0.36108	0.09741
Torsin-3A	Tor3a	Q9ER38	-0.36013	0.00005
Odorant-binding protein 1a	Obp1a	Q9D3H2	-0.35845	0.22990
G-protein coupled receptor family C group 5 member D	Gprc5d	Q9JIL6	-0.34793	0.13028
Ubiquitin-like protein ISG15	Isg15	Q64339	-0.34678	0.15813
Keratin, type II cytoskeletal 8	Krt8	P11679	-0.34429	0.52322
Vang-like protein 1	Vangl1	Q80Z96	-0.34181	0.04582
THAP domain-containing protein 11	Thap11	Q9JJD0	-0.34115	0.05393
Nucleolar protein 16	Nop16	Q9CPT5	-0.32729	0.08143
Keratin, type I cuticular Ha1	Krt31	Q61765	-0.32586	0.17668
A-kinase anchor protein 10, mitochondrial	Akap10	O88845	-0.32559	0.01312
Interferon-induced protein 44-like	Ifi44l	Q9BDB7	-0.31835	0.19422
Secretoglobin family 2B member 2	Scgb2b2	Q6UGQ3	-0.31666	0.33570
Hemoglobin subunit alpha =2	Hba	P01942	-0.31113	0.07083
Tenomodulin	Tnmd	Q9EP64	-0.30269	0.13153
Hemoglobin subunit beta-2	Hbb-b2	P02089	-0.30260	0.16145
Keratin-associated protein 7-1	Krtap7-1	Q9D3I6	-0.29986	0.06255
Polyunsaturated fatty acid lipoxygenase ALOX8	Alox8	O35936	-0.28896	0.14990
Oligosaccharyltransferase complex subunit OSTC	Ostc	Q78XF5	-0.28665	0.04605
Keratin, type II cuticular Hb5	Krt85	Q9Z2T6	-0.28589	0.23906
ATP-binding cassette sub-family C member 9	Abcc9	P70170	-0.28455	0.48742
Hemoglobin subunit beta-1	Hbb-b1	P02088	-0.28285	0.10200
E3 ubiquitin-protein ligase KCMF1	Kcmf1	Q80UY2	-0.27988	0.44668
Myelin proteolipid protein	Plp1	P60202	-0.27615	0.37613

CB1 cannabinoid receptor-interacting protein 1	Cnrip1	Q5M8N0	-0.27467	0.02494
Serum amyloid P-component	Apcs	P12246	-0.27045	0.14013
H-2 class I histocompatibility antigen, L-D alpha chain	H2-L	P01897	-0.26998	0.04122
Ferroxidase HEPHL1	Hephl1	Q3V1H3	-0.26967	0.17757
Keratin, type I cytoskeletal 16	Krt16	Q9Z2K1	-0.26874	0.42289
Keratin, type II cytoskeletal 6A	Krt6a	P50446	-0.26801	0.28358
Acyl-CoA desaturase 1	Scd1	P13516	-0.26747	0.00475
Interferon-induced protein with tetratricopeptide repeats 3	Ifit3	Q64345	-0.26622	0.11035
Keratin, type II cytoskeletal 6B	Krt6b	Q9Z331	-0.26560	0.53080
Keratin, type II cytoskeletal 72	Krt72	Q6IME9	-0.26542	0.23331
Keratin, type II cuticular Hb4	Krt84	Q99M73	-0.26471	0.07547
UPF0449 protein C19orf25 homolog	/	Q9D7E4	0.26365	0.03929
Haloacid dehalogenase-like hydrolase domain-containing 5	Hdhd5	Q91WM2	0.26457	0.37094
Rootletin	Crocc	Q8CJ40	0.27597	0.09549
Troponin T, slow skeletal muscle	Tnnt1	O88346	0.27728	0.08731
Zinc finger protein basonuclin-2	Bnc2	Q8BMQ3	0.27892	0.10682
Seminal vesicle secretory protein 4	Svs4	P18419	0.28155	0.14199
Kinesin-like protein KIF13A	Kif13a	Q9EQW7	0.28386	0.06780
Phosphofurin acidic cluster sorting protein 2	Pacs2	Q3V3Q7	0.28586	0.05661
Cytosolic phospholipase A2 epsilon	Pla2g4e	Q50L42	0.28875	0.27782
Gamma-tubulin complex component 2	Tubgcp2	Q921G8	0.28963	0.04011
Lipolysis-stimulated lipoprotein receptor	Lsr	Q99KG5	0.29875	0.06916
Eukaryotic translation initiation factor 2 subunit 3, Y-linked	Eif2s3y	Q9Z0N2	0.30223	0.15176
Nuclear receptor-binding factor 2	Nrbf2	Q8VCQ3	0.30451	0.00157
Ubiquitin thioesterase OTU1	Yod1	Q8CB27	0.31073	0.01312
Heat shock 70 kDa protein 12B	Hspa12b	Q9CZJ2	0.31172	0.01049
Anillin OS=Mus musculus	Anln	Q8K298	0.31463	0.24838
Protein FAM91A1	Fam91a1	Q3UVG3	0.31986	0.21999
FHF complex subunit HOOK interacting protein 1A	Fhip1a	Q505K2	0.32544	0.10872
InaD-like protein	Patj	Q63ZW7	0.34107	0.00148
Cdc42 effector protein 5	Cdc42ep5	Q9Z0X0	0.34983	0.40324
Cyclin-dependent kinase 4	Cdk4	P30285	0.36038	0.45719
FAST kinase domain-containing protein 4	Tbrg4	Q91YM4	0.36279	0.07446
E3 ubiquitin-protein ligase DTX3L	Dtx3l	Q3UIR3	0.39155	0.36608
Protein N-terminal glutamine	Ntaq1	Q80WB5	0.40577	0.07478

amidohydrolase				
Serine/threonine-protein kinase RIO1	Riok1	Q922Q2	0.43559	0.17357
Phospholipase DDHD2	Ddhd2	Q80Y98	0.46772	0.02026
Disintegrin and metalloproteinase domain-containing protein 17	Adam17	Q9Z0F8	0.50090	0.06697
RasGAP-activating-like protein 1	Rasal1	Q9Z268	0.52002	0.00421
Dehydrogenase/reductase SDR family member on chromosome X homolog	Dhrsx	Q8VBZ0	0.52449	0.06761
Platelet-activating factor acetylhydrolase 2, cytoplasmic	Pafah2	Q8VDG7	0.55420	0.18817
Protein mono-ADP-ribosyltransferase PARP4	Parp4	E9PYK3	0.59002	0.37570
Uricase	Uox	P25688	0.60856	0.56363
Ribokinase	Rbks	Q8R1Q9	0.65071	0.37526
Polyphosphoinositide phosphatase	Fig4	Q91WF7	0.65380	0.19794
Major urinary protein 3	Mup3	P04939	0.70052	0.23418
Major urinary protein 17	Mup17	B5X0G2	0.95580	0.11504
Major urinary protein 2	Mup2	P11589	0.99134	0.19731
Major urinary protein 1	Mup1	P11588	1.36602	0.12425

Table S4. Proteins of Trusted proteins in skin between MC and BC (Fold change ≥ 1.2 or Fold change ≤ 1/1.2).

Protein Name	Gene Name	UniProt ID	LogFC	P.values
Repetin	Rptn	P97347	-1.32120	0.05716
Small proline-rich protein 2D	Sprr2d	O70555	-1.25945	0.00285
Interleukin-36 alpha	Il36a	Q9JLA2	-1.23494	0.00429
Major urinary protein 1	Mup1	P11588	-1.01035	0.04732
Cornifin-B	Sprr1b	Q62267	-0.98086	0.01626
Cdc42 effector protein 5	Cdc42ep5	Q9Z0X0	-0.97232	0.04995
Polyunsaturated fatty acid lipoxygenase ALOX8	Alox8	O35936	-0.84881	0.01926
Major urinary protein 17	Mup17	B5X0G2	-0.81780	0.03793
Insulin receptor-related protein	Insrr	Q9WTL4	-0.77527	0.34749
Protein S100-A9	S100a9	P31725	-0.77261	0.06355
Chitinase-3-like protein 1	Chi3l1	Q61362	-0.72435	0.04542
Major urinary protein 2	Mup2	P11589	-0.72366	0.08720
Neutrophilic granule protein	Ngp	O08692	-0.68083	0.05195
Protein S100-A8	S100a8	P27005	-0.66909	0.04279
Filaggrin (Fragment)	Flg	P11088	-0.66019	0.06776
Beta-defensin 6	Defb6	Q91VD6	-0.65019	0.00709
Stefin-3	Stfa3	P35173	-0.59339	0.04580
Serine protease 27	Prss27	Q8BJR6	-0.54254	0.02442
LIM domain-containing protein 2	Limd2	Q8BGB5	-0.53273	0.16534
Centrosomal protein CCDC61	Ccdc61	Q3UJV1	-0.52664	0.00262

NADH-ubiquinone oxidoreductase chain 2	mt-Nd2	P03893	-0.52283	0.05550
Zinc finger protein 865	Znf865	Q3U3I9	-0.51102	0.01614
Glutaredoxin-1	Glrx	Q9QUH0	-0.50641	0.04893
U1 small nuclear ribonucleoprotein C	Snrpc	Q62241	-0.48051	0.01118
Isochorismatase domain-containing protein 2A	Isoc2a	P85094	-0.47313	0.10965
Transmembrane protein 14C	Tmem14c	Q9CQN6	-0.47286	0.02886
Major urinary protein 3	Mup3	P04939	-0.44858	0.16736
Myoglobin	Mb	P04247	-0.44370	0.08915
N-acetylgalactosamine kinase	Galk2	Q68FH4	-0.44350	0.01482
Cyclin-T2	Ccnt2	Q7TQK0	-0.42832	0.07144
NADH-ubiquinone oxidoreductase chain 1	Mtnd1	P03888	-0.42522	0.02524
Prostaglandin E synthase	Ptges	Q9JM51	-0.40536	0.06421
Caspase-14	Casp14	O89094	-0.39060	0.02788
Cytochrome c oxidase subunit 7A2, mitochondrial	Cox7a2	P48771	-0.38927	0.01638
Cytochrome b	Mt-Cyb	P00158	-0.38421	0.02704
Murinoglobulin-1	Mug1	P28665	-0.37914	0.04909
Low affinity immunoglobulin gamma Fc region receptor III	Fcgr3	P08508	-0.37906	0.01677
Lactotransferrin	Ltf	P08071	-0.37758	0.05270
PRKC apoptosis WT1 regulator protein	Pawr	Q925B0	-0.37747	0.05345
High mobility group nucleosome-binding domain-containing protein 3	Hmgn3	Q9DCB1	-0.37382	0.02300
Sodium/glucose cotransporter 1	Slc5a1	Q8C3K6	-0.37019	0.06936
Tapasin-related protein	Tapbpl	Q8VD31	-0.36892	0.37303
Leptin receptor gene-related protein	Leprot	O89013	-0.36708	0.07831
Myeloperoxidase	Mpo	P11247	-0.36674	0.01173
60S ribosomal protein L35	Rpl35	Q6ZWV7	-0.36564	0.04420
Serine protease inhibitor A3K	Serpina3k	P07759	-0.36079	0.03511
Cytochrome c oxidase subunit NDUFA4	Ndufa4	Q62425	-0.35854	0.12794
Seminal vesicle secretory protein 4	Svs4	P18419	-0.35839	0.08775
Cytochrome c oxidase subunit 6A1, mitochondrial	Cox6a1	P43024	-0.35738	0.01065
3-beta-hydroxysteroid-Delta(8),Delta(7)-isomerase	Ebp	P70245	-0.35680	0.00391
Vitamin K epoxide reductase complex subunit 1	Vkorc1	Q9CRC0	-0.35272	0.12525
Arginase-1	Arg1	Q61176	-0.35262	0.32047
Acid sphingomyelinase-like phosphodiesterase 3a	Smpdl3a	P70158	-0.35219	0.00040
Serine/threonine-protein kinase ATR	Atr	Q9JKK8	-0.35181	0.02347
Zinc finger MYM-type protein 2	Zmym2	Q9CU65	-0.35132	0.15783
Thymosin beta-10	Tmsb10	Q6ZWY8	-0.35118	0.18368
Putative peptidyl-tRNA hydrolase PTRHD1	Ptrhd1	D3Z4S3	-0.34639	0.05284
Gasdermin-C	Gsdmc	Q99NB5	-0.34439	0.15433
Glycerol kinase	Gk	Q64516	-0.34365	0.01657

ATP synthase subunit ATP5MPL, mitochondrial	Atp5mpl	P56379	-0.34337	0.07887
Transmembrane protein 41B	Tmem41b	Q8K1A5	-0.34223	0.00831
Calreticulin	Calr	P14211	-0.34190	0.06704
TRAF-type zinc finger domain-containing protein 1	Trafd1	Q3UDK1	-0.34033	0.31849
Glucose-6-phosphate exchanger SLC37A2	Slc37a2	Q9WU81	-0.33908	0.03977
Dehydrogenase/reductase SDR family member 7C	Dhrs7c	Q8CHS7	-0.33454	0.33543
Acyl-coenzyme A thioesterase 13	Acot13	Q9CQR4	-0.33336	0.07306
ATP synthase membrane subunit K, mitochondrial	Atp5mk	Q78IK2	-0.33269	0.07003
Solute carrier organic anion transporter family member 1A1	Slco1a1	Q9QXZ6	-0.33201	0.17202
Thymosin beta-4	Tmsb4x	P20065	-0.32934	0.09989
Polyribonucleotide 5'-hydroxyl-kinase Clp1	Clp1	Q99LI9	-0.32257	0.50562
Synaptic vesicle glycoprotein 2C	Sv2c	Q69ZS6	-0.32150	0.02321
NGFI-A-binding protein 2	Nab2	Q61127	-0.31685	0.11095
Nucleoporin Nup43	Nup43	P59235	-0.31545	0.26700
Activating molecule in BECN1-regulated autophagy protein 1	Ambra1	A2AH22	-0.31397	0.08915
Phospholipase DDHD2	Ddhd2	Q80Y98	-0.31217	0.02742
ADP-ribosylation factor-like protein 6-interacting protein 4	Arl6ip4	Q9JM93	-0.31117	0.10193
Y-box-binding protein 1	Ybx1	P62960	-0.31112	0.09388
39S ribosomal protein L12, mitochondrial	Mrpl12	Q9DB15	-0.31077	0.00999
Phosphofurin acidic cluster sorting protein 2	Pacs2	Q3V3Q7	-0.31123	0.05661
Glutathione S-transferase Mu 4	Gstm4	Q8R5I6	-0.29946	0.37750
Rho guanine nucleotide exchange factor 18	Arhgef18	Q6P9R4	-0.29843	0.02163
SPRY domain-containing SOCS box protein 2	Spsb2	O88838	-0.29758	0.00190
Prothymosin alpha	Ptma	P26350	-0.29498	0.03184
Transient receptor potential cation channel subfamily M member 7	Trpm7	Q923J1	-0.29399	0.32487
Vasorin	Vasn	Q9CZT5	-0.29274	0.12629
Involucrin	Ivl	P48997	-0.28984	0.00940
Keratin, type I cytoskeletal 16	Krt16	Q9Z2K1	-0.28979	0.44332
Tyrosine-protein kinase BTK	Btk	P35991	-0.28843	0.05958
Interferon-inducible GTPase 1	Iigp1	Q9QZ85	-0.28784	0.30825
Intraflagellar transport protein 140 homolog	Ift140	E9PY46	-0.28707	0.03649
Nurim	Nrm	Q8VC65	-0.28699	0.00192
Chromodomain Y-like protein 2	Cdyl2	Q9D5D8	-0.28608	0.26104
Cornifin-A	Sprr1a	Q62266	-0.28478	0.03858
NADH dehydrogenase [ubiquinone] flavoprotein	Ndufv3	Q8BK30	-0.28477	0.07683

3, mitochondrial				
Probable tRNA (uracil-O(2)-)-methyltransferase	Trmt44	Q9D2Q2	-0.28108	0.02507
Alpha-actinin-3	Actn3	O88990	-0.28100	0.16148
Stefin-1	Stfa1	P35175	-0.28021	0.25389
Dynein light chain roadblock-type 1	Dynlrb1	P62627	-0.27965	0.04271
CDP-diacylglycerol--inositol 3-phosphatidyltransferase	Cdipt	Q8VDP6	-0.27801	0.06824
Mitochondrial fission 1 protein	Fis1	Q9CQ92	-0.27666	0.09273
m7GpppN-mRNA hydrolase	Dcp2	Q9CYC6	-0.27612	0.03090
NADH-ubiquinone oxidoreductase chain 5	Mtnd5	P03921	-0.27576	0.06021
Protein FAM162A	Fam162a	Q9D6U8	-0.27469	0.02953
Nuclear receptor-binding factor 2	Nrbf2	Q8VCQ3	-0.27245	0.00799
Apolipoprotein A-II	Apoa2	P09813	-0.27200	0.06764
Zinc finger protein basonuclin-2	Bnc2	Q8BMQ3	-0.27179	0.04654
Microsomal glutathione S-transferase 1	Mgst1	Q91VS7	-0.27171	0.08888
Superoxide dismutase [Mn], mitochondrial	Sod2	P09671	-0.27064	0.05566
Zinc finger protein 706	Znf706	Q9D115	-0.26932	0.00621
ATP synthase-coupling factor 6, mitochondrial	Atp5pf	P97450	-0.26904	0.08897
Interleukin-36 beta	Il36b	Q9D6Z6	-0.26676	0.06795
Ras-interacting protein 1	Rasip1	Q3U0S6	-0.26642	0.00905
Kallikrein-1	Klk1	P15947	-0.26624	0.48929
V-type proton ATPase 16 kDa proteolipid subunit c	Atp6v0c	P63082	-0.26517	0.04937
Keratin, type I cytoskeletal 13	Krt13	P08730	0.26430	0.08659
Dystonin	Dst	Q91ZU6	0.26497	0.00331
Vomeromodulin	Bpifb9a	Q80XI7	0.26771	0.00926
Secretoglobin family	Scgb2b20	Q9JI02	0.26799	0.12458
Keratin, type II cuticular 87	Krt87	Q6IMF0	0.26803	0.24727
Keratin, type II cytoskeletal 80	Krt80	Q0VBK2	0.26987	0.01604
MAM domain-containing protein 2	Mamdc2	Q8CG85	0.27160	0.00246
Keratin, type II cuticular Hb6	Krt86	P97861	0.27323	0.25496
Odorant-binding protein 2a	Obp2a	Q8K1H9	0.27475	0.10890
Non-homologous end-joining factor 1	Nhej1	Q3KNJ2	0.27608	0.29525
Cytochrome P450 2B10	Cyp2b10	P12791	0.27734	0.03125
Keratin, type I cuticular Ha4	Krt34	Q9D646	0.27902	0.31634
Keratin-associated protein 6-5	Krtap6-5	Q925H3	0.27993	0.21757
Gamma-tubulin complex component 2	Tubgcp2	Q921G8	0.27992	0.01305
Protein phosphatase PTC7 homolog	Pptc7	Q6NVE9	0.28545	0.53432
Desmoglein-1-alpha	Dsg1a	Q61495	0.28593	0.01942
Cytochrome P450 4B1	Cyp4b1	Q64462	0.28678	0.00708
Keratin-associated protein 5-5	Krtap5-5	Q2TA51	0.28793	0.44164
Filaggrin-2	Flg2	Q2VIS4	0.28862	0.02113
Keratinocyte proline-rich protein	Kprp	B2RUR4	0.28978	0.01369

Metallothionein-4	Mt4	P47945	0.29137	0.03136
Vang-like protein 1	Vangl1	Q80Z96	0.29146	0.04582
Desmocollin-3	Dsc3	P55850	0.29317	0.01162
Adenylate cyclase type 8	Adcy8	P97490	0.29462	0.32208
Ephrin type-A receptor 4	Epha4	Q03137	0.29745	0.01226
Myocilin	Myoc	O70624	0.29793	0.01394
Dual specificity protein phosphatase 14	Dusp14	Q9JLY7	0.29791	0.06769
Loricrin	Loricrin	P18165	0.31101	0.08398
FHF complex subunit HOOK interacting protein 1A	Fhip1a	Q505K2	0.31769	0.00712
Keratin-associated protein 5-2	Krtap5-2	Q9D5Z7	0.32425	0.54177
Interferon-induced protein with tetratricopeptide repeats 1	Ifit1	Q64282	0.32624	0.13829
Serine/threonine-protein kinase WNK4	Wnk4	Q80UE6	0.32952	0.02410
Protein yippee-like 5	Ypel5	P62700	0.33780	0.11913
Ig heavy chain V region VH558 A1/A4	Gm5629	P06327	0.34256	0.23467
Gamma-butyrobetaine dioxygenase	Bbox1	Q924Y0	0.34278	0.01207
Putative transferase CAF17 homolog, mitochondrial	Iba57	Q8CAK1	0.34394	0.06689
Ig heavy chain V-III region T957	/	P01800	0.34413	0.26008
Disintegrin and metalloproteinase domain-containing protein 17	Adam17	Q9Z0F8	0.34525	0.15697
Troponin T, slow skeletal muscle	Tnnt1	O88346	0.35376	0.00398
N-acetylneuraminate lyase	Npl	Q9DCJ9	0.35424	0.02781
Alpha-1,6-mannosyl-glycoprotein 1-beta-N-acetylglucosaminyltransferase	Mgat1	Q921V5	0.35426	0.09741
Keratin, type I cytoskeletal 24	Krt24	A1L317	0.35881	0.00589
Immunoglobulin kappa constant	Igkc	P01837	0.36099	0.16975
Polyunsaturated fatty acid (12S)/(13S)-lipoxygenase, epidermal-type	Alox12e	P55249	0.37089	0.01830
Tissue factor	F3	P20352	0.37696	0.00924
FYVE, RhoGEF and PH domain-containing protein 4	Fgd4	Q91ZT5	0.38005	0.10449
Phosphatidate cytidylyltransferase 2	Cds2	Q99L43	0.39257	0.02149
Ig gamma-3 chain C region		P03987	0.41808	0.03716
Paired amphipathic helix protein Sin3b	Sin3b	Q62141	0.44475	0.57687
Plexin-A4	Plxna4	Q80UG2	0.46060	0.04424
Keratin-associated protein 21-1	Krtap21-1	Q925H4	0.47485	0.07867
Secretoglobin family 2B member 24	Scgb2b24	Q7M747	0.51468	0.03769
Acyl-CoA desaturase 1	Scd1	P13516	0.46747	0.00475
Keratin-associated protein 6-2	Krtap6-2	O08884	0.52512	0.14274
Keratin, type II cytoskeletal 2 oral	Krt76	Q3UV17	0.58489	0.45623
Ig heavy chain V region 3-6	Ighv3-6	P18531	0.58909	0.02869
Odorant-binding protein 1a	Obp1a	Q9D3H2	0.67417	0.02976

Keratin, type II cytoskeletal 1b	Krt77	Q6IFZ6	0.68593	0.13743
Secretoglobin family 2B member 2	Scgb2b2	Q6UGQ3	0.69397	0.02362
Tropomodulin-1	Tmod1	P49813	0.72707	0.11263
Ig gamma-2A chain C region, membrane-bound form	Igh-1a	P01865	0.80559	0.09882
Ribokinase	Rbks	Q8R1Q9	0.87657	0.13249
